# Beluga whale and bottlenose dolphin ACE2 proteins allow cell entry mediated by spike protein from three variants of SARS-CoV-2

**DOI:** 10.1098/rsbl.2023.0321

**Published:** 2023-12-06

**Authors:** H. M. Stone, E. Unal, T. A. Romano, P. E. Turner

**Affiliations:** ^1^ Graduate Program in Microbiology, Yale School of Medicine, New Haven, CT 06520, USA; ^2^ Department of Ecology and Evolutionary Biology, Yale University, New Haven, CT 06520, USA; ^3^ Sea Research Foundation, Inc. d/b/a Mystic Aquarium, Mystic, CT 06355, USA; ^4^ Department of Marine Sciences, University of Connecticut Avery Point Campus, Groton, CT 06340, USA

**Keywords:** SARS-CoV-2, *Tursiops truncatus*, *Delphinapterus leucas*, host-range

## Abstract

Severe acute respiratory syndrome coronavirus 2 (SARS-CoV-2) viruses infect numerous non-human species. Spillover of SARS-CoV-2 into novel animal reservoirs may present a danger to host individuals of these species, particularly worrisome in populations already endangered or threatened by extinction. In addition, emergence in new reservoirs could pose spillback threats to humans, especially in the form of virus variants that further mutate when infecting other animal hosts. Previous work suggests beluga whales (*Delphinapterus leucas*) and bottlenose dolphins (*Tursiops truncatus*) may be at risk owing to their formation of social groups, contact with humans, exposure to contaminated wastewater, and structure of their angiotensin-converting enzyme 2 (ACE2) proteins, which SARS-CoV-2 uses as a cellular receptor. We examined marine-mammal susceptibility to virus infection by challenging 293T cells expressing beluga or dolphin ACE2 with pseudovirions bearing the SARS-CoV-2 spike protein. Beluga and dolphin ACE2 were sufficient to allow cell entry by an early pandemic isolate (Wuhan-Hu-1) and two evolved variants (Delta B.1.617.2 and Omicron BA.1 strains). We conclude that SARS-CoV-2 poses a potential threat to marine mammal reservoirs that should be considered in surveillance efforts.

## 1. Introduction

Severe acute respiratory syndrome coronavirus 2 (SARS-CoV-2) has a probable zoonotic origin [1,2]. SARS-CoV-2 infections have been detected in a variety of non-human animals, including domesticated cats [3], white-tailed deer [4], captive tigers [5], certain monkey species [6], Antillean manatees [7] and captive hippopotami [8].

Host range specificity of SARS-CoV-2 is largely attributed to interactions between the virus's spike protein and its mammalian-cell receptor, angiotensin-converting enzyme 2 (ACE2) [9]. During SARS-CoV-2 infection, binding of virus particles to ACE2 allows their entry into host cells. Other cell-surface proteins such as transmembrane serine protease 2 assist with key steps of the virus infection process, such as priming of the spike protein [10]. Several computational studies predict susceptibility of various animal species to SARS-CoV-2 based on their ACE2 structure [11–13] with correlations drawn between prediction results and known infectable species [14]. Further studies experimentally assess ACE2 orthologues for compatibility with spike using cell-binding and entry assays [9,15–19]. Surprisingly, taxonomy is not always predictable of ACE2 affinity to spike protein [20] and closely related species still display extensive variation in susceptibility to SARS-CoV-2 infection [16].

The threat of SARS-CoV-2 to marine mammals such as cetaceans may be particularly significant. Computational studies based on ACE2 structure predict high susceptibility of bottlenose dolphin (*Tursiops truncatus*) and beluga whale (*Delphinapterus leucas*) species for infection by SARS-CoV-2 [11–13], with one study predicting greater infection-susceptibility in dolphins relative to humans [13]. Novel coronaviruses were discovered previously in both dolphins [21] and belugas [22]. One experimental study finds that wild-type spike is able to enter cells expressing beluga and dolphin ACE2, although at a lower efficiency than cells expressing human ACE2 [15].

Dolphins and belugas typically live in social groups that may foster virus transmission between infected and uninfected individuals. Also, these animals may come into contact with humans during encounters in the wild and when hunted by indigenous communities. Human-to-cetacean transmission in aquariums and stranding or rehabilitation centres is of particular concern, given the day-to-day contact of veterinary and husbandry personnel with the animals. Also, concern has been raised about the potential for infectious SARS-CoV-2 present in wastewater to be transmissible to marine life [13]. Although transmission via wastewater should be low probability owing to dilution and/or decay of virus particles [23,24], one study finds that viruses at initially high titer may still be viable after 20 days in artificial seawater and up to 12 days on plastic bags at 4°C [25]. Given the prevalence of plastic pollution in the ocean and the cold temperatures in which beluga whales in particular can thrive, circumstances may favour the possibility of transmission from the abiotic environment or fomites.

As SARS-CoV-2 continues to evolve, so too does its host range [26]. In the present study, we explored susceptibility of cells expressing ACE2 from bottlenose dolphins and beluga whales to entry by an early-pandemic viral isolate (strain Wuhan-Hu-1) and by the evolved Delta (B.1.617.2) and Omicron (BA.1) variants.

## 2. Methods

### (a) Generation of angiotensin-converting enzyme 2 plasmids

Pieces (1 cm^3^) of organs from dolphin (lung and kidney) and beluga (kidney) were collected during necropsy as part of the comprehensive end of life care of Navy marine mammals under 10 USC Sec 7524 and SECNAVINST 3900.41H which directs that Navy marine mammals be provided the highest quality of care. The U.S. Navy Marine Mammal Program (MMP) is accredited by AAALAC International and adheres to the national standards of the U.S. Public Health Service Policy on the Humane Care and Use of Laboratory Animals and the Animal Welfare Act. Samples were archived in liquid nitrogen until RNA extraction, following the protocol in [27]. Briefly, up to 100 mg of each tissue was manually ground into a powder with a sterile mortar and pestle containing liquid nitrogen. The powder was then transferred into PureZOL^TM^ RNA isolation reagent within an Aurum^TM^ total RNA fatty and fibrous tissue kit (Bio-Rad), and homogenized using a hand-held homogenizer (Omni International). RNA extractions were performed per the manufacturer's protocol including a genomic DNA removal step. RNA concentration and purity (A260/280) were measured using a BioTek epoch microplate spectrophotometer, and RNA integrity was assessed using agarose gel electrophoresis [28]. Human RNA was extracted from colorectal adenocarcinoma-derived Caco-2 cells (American Type Culture Collection [ATCC] no. HTB-37) using the RNeasy Kit (Qiagen).

RNA was converted into complementary DNA (cDNA) using an iScript select cDNA synthesis kit (Bio-Rad). The code-determining sequence of each species' ACE2 was polymerase chain reaction (PCR)-amplified using iProof high fidelity DNA polymerase (Bio-Rad). ACE2 sequences were substituted into vector plasmid pCAGGS-mCherry (Addgene no. 41583, a gift from Phil Sharp, [29]) in place of mCherry, with the Kozak sequence preserved and C-terminal FLAG-tags (sequence DYKDDDDK) added, using a Gibson assembly cloning kit (New England Biolabs). ACE2 sequences of engineered plasmids were verified via Sanger sequencing and compared to NCBI reference transcripts. Beluga ACE2 was consistent with the available transcript (NCBI reference sequence: XM_022562652.2) as was human ACE2 (NCBI reference sequence: NM_021804.3). However, a G→A base change was noted between dolphin ACE2 and the available transcript (NCBI reference sequence: XM_019925618.2) that resulted in amino acid substitution A20T. The same sequence was observed in cDNA made from RNA extracted from two different tissue sources (kidney versus lung) obtained from two different dolphins.

### (b) Cell culture

HEK293T human-kidney-derived cells (ATCC no. CRL-3216) were cultured at 37°C and 5% CO_2_ in complete medium consisting of Dulbecco's modified eagle medium (Gibco) with 10% fetal bovine serum (Gibco) and working concentration of penicillin-streptomycin (100 U ml^−1^, Gibco).

### (c) Generation of pseudoviruses

A previously published protocol [30] was followed. First, 3–5 × 10^6^ 293T cells were seeded into 10 cm dishes. The next day, cells were transfected with the appropriate spike plasmid (Wuhan-Hu-1: BEI NR-52310; Delta: Addgene no. 185593, a gift from Marceline Côté, [31]; Omicron: Addgene no. 185452, a gift from Marceline Côté, [32]) using the calcium-phosphate profection mammalian transfection system (Promega). All spike plasmids were human-codon optimized and coded for the full-length protein. There were 20 µg of plasmid used per dish. Approximately 20 h later, media was removed and cells were infected with 50 µl vesicular stomatitis virus strain VSVΔG-Rluc decorated with VSV-G (seed particles obtained from Benhur Lee, Icahn School of Medicine at Mt Sinai; stocks renewed using pCMV-VSV-G, Addgene no. 8454, a gift from Bob Weinberg, [33]) in 5 ml media. After 1 h incubation, cells were washed with phosphate-buffered saline (PBS) and 8 ml fresh media containing 1.6 µg of antibody neutralizing against VSV-G (Millipore Sigma no. MABF2337-25UG) were added. The supernatant was harvested 24 h post infection and aliquots were stored at −80°C.

### (d) Angiotensin-converting enzyme 2 transfection, infection, and luminescence assays

Approximately 10 000 293T cells were seeded per well of a white-walled 96-well plate (Thermo-Scientific Nunc), previously coated in Geltrex LDEV-free reduced growth factor basement membrane matrix (Gibco) according to the manufacturer's thin layer non-gelling method to enhance cell adhesion. The next day, the cells were transfected using the calcium-phosphate profection mammalian transfection system (Promega). Transfection solution was prepared with the appropriate ACE2 plasmid following manufacturer instructions for 10 µg plasmid per 10 cm dish. To proportionally reduce volume added according to relative surface area, 4 µl of solution were added per well of the 96-well plate. Then, 24 h after transfection, the cells were infected with pseudovirus following a previously published protocol [30]. The spent medium was removed, and 100 µl of fresh media containing the indicated dilution of pseudovirus was added per well. Plates were spinoculated for one hour at 330 *g*. At 18–24 h post infection, media was removed, cells were washed with PBS, and cells were lysed using Renilla luciferase assay system lysis buffer (Promega) and one freeze–thaw cycle. The lysate was assayed for luminescence using the Renilla luciferase assay system (Promega) on a Synergy H1 microplate reader (BioTek).

### (e) Western blots

The same ACE2 transfection protocol described above was followed, with the modification that 96-well clear-walled plates were used (Falcon). At 24 h post transfection, cells were washed in PBS, lysed in Laemmli sample buffer with added β-mercaptoethanol (Bio-Rad), and heated at 100°C for 5 min. Lysates were stored at −80°C until further processing. Lysates were run on a 7.5% mini-PROTEAN TGX gel (Bio-Rad). For the Western blot, primary antibodies were used against the FLAG-tag at 1 : 8000 dilution (ProteinTech no. 20543-1-AP) or against GAPDH at 1 : 2500 dilution (Novus Biologicals no. NB300-327-0.025 ml). Horseradish peroxidase anti-rabbit was used at 1 : 5000 dilution (Biotium no. 20402) as the secondary antibody. Blots were visualized using Clarity ECL Western blotting substrate (Bio-Rad) and imaged on a ChemiDoc touch imaging system (Bio-Rad).

### (f) Statistics and graphs

Analysis was conducted in R (version 4.2.3, [34]) using packages *data.table* [35], *ggplot2* [36], *dplyr* [37], *rstatix* [38] and *FSA* [39]. For statistical analysis, data were analysed using Welch's *t*-test with Bonferroni correction unless otherwise noted. Graphs were created in R and aesthetically modified (e.g. significance asterisks added) in Adobe Illustrator 2023.

## 3. Results and discussion

We investigated whether ACE2 proteins of the beluga whale (*D. leucas*) and the bottlenose dolphin (*T. truncatus*) could each allow cell entry via the spike of different SARS-CoV-2 variants. To do so, we first transfected human 293T cells with a mammalian overexpression vector containing the coding sequence for human (hACE2), beluga (bACE2), or dolphin (dACE2) ACE2 protein.

**Figure 1.**
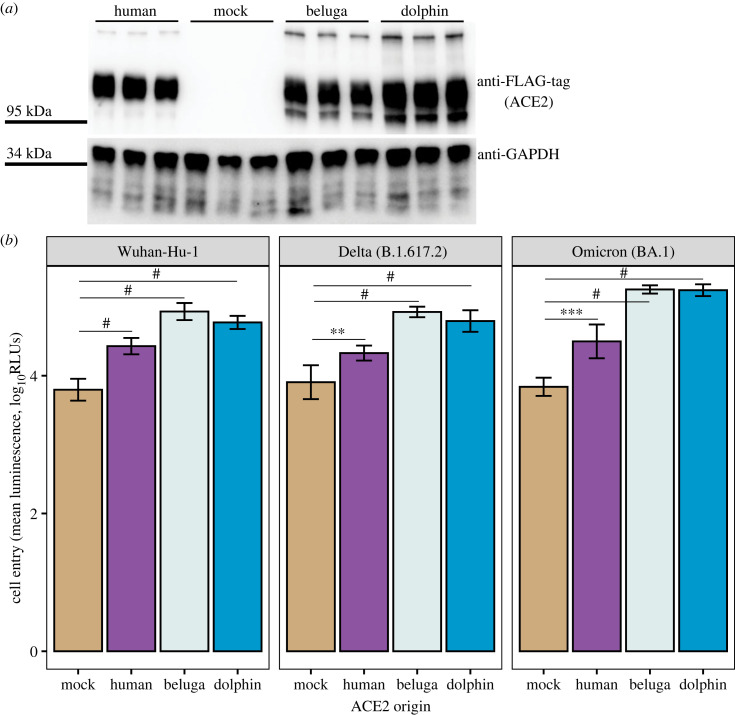
(*a*) Western blot of 293T cells transfected with FLAG-tagged ACE2 of human, beluga, or dolphin origin, or with an empty vector (mock). Each lane represents cell lysate collected from a separate plate well. The top panel shows lysate blotted against anti-FLAG-tag, and the bottom panel shows lysate from the same samples blotted against anti-GAPDH as the loading control. Top and bottom panels were imaged separately. (*b*) Cell entry, indicated by luminescence, mediated by three spike variants into 293T cells transfected with ACE2 of human, beluga, or dolphin origin. Mock represents 293T cells transfected with an empty vector. Cells were challenged with VSVΔG-Rluc pseudoviruses decorated with spike protein from Wuhan-Hu-1 (left panel), Delta (middle), or Omicron (right). Increasing luminescence readings (in relative luminescence units (RLUs)) indicate increasing cell entry. Each point represents log_10_ of the average luminescence readout for eight total wells pooled from two separate experiments (four wells per experiment). Different variants were assayed separately. Error bars represent mean ± one standard deviation. **p < 0.01, ***p < 0.001, ^#^*p* < 0.0001 for Welch's *t*-test with a Bonferroni correction for three comparisons. Statistical comparisons were made only between mock and each ACE2 type.

We confirmed ACE2 expression using Western blots. We blotted cells transfected with FLAG-tagged ACE2 against an anti-FLAG antibody, demonstrating positive expression across plasmids (figure 1*a*).

To test SARS-CoV-2 susceptibility of the two marine mammals, we challenged the ACE2-transfected cells with VSVΔG-Rluc pseudovirus particles decorated with spike protein from either the Wuhan-Hu-1, Delta (B.1.617.2), or Omicron (BA.1) strains. We tested three five-fold dilutions of each pseudovirus and observed similar trends across each dilution. For simplicity, only the most concentrated dilution (1 : 200) is shown in figure 1*b*, with the complete dilution series and associated statistical results depicted in the electronic supplementary material, figure S1 and table S1. For all three spike variants at the 1 : 200 dilution, we observed a significant increase in luminescence in cells expressing beluga and dolphin ACE2 relative to cells transfected with the empty vector (table 1). Because the recombinant pseudoviruses contain the gene for Renilla luciferase, luminescence is used as an indicator of virus cell entry.

**Table 1.** Adjusted *p*-values for Welch's *t*-tests on log_10_ transformed RLU values with Bonferroni correction for three comparisons. (Each virus variant was analysed separately; rows indicate outcomes from an individual test. Separate experiments were pooled for this analysis. **p < 0.01, ***p < 0.001, ^#^*p* < 0.0001.)

virus	dilution	beluga versus mock	dolphin versus mock	human versus mock
Wuhan-Hu-1	1 : 200	1.53 × 10^−9^^#^	2.15 × 10^−8^^#^	1.76 × 10^−6^^#^
Delta	1 : 200	7.86 × 10^−6^^#^	5.7 × 10^−6^^#^	3 × 10^−3^ **
Omicron	1 : 200	4.11 × 10^−10^^#^	2.73 × 10^−11^^#^	1.13 × 10^−4^ ***

We concluded that, based on ACE2 mediated cell entry, belugas and dolphins appeared to be species susceptible to infection by not only an early pandemic viral isolate but also two evolved variants of SARS-CoV-2. Prior studies indicate that novel variants of SARS-CoV-2 probably have altered host ranges [18,19,26,40]. Although some previously susceptible animal species seem to be resistant to variants such as Omicron BA.1 based on ACE2 binding data [19], the reverse trend wherein BA.1 appears to bind ACE2 from a broader range of species has also been observed [18]. One study suggests that multiple mutations to spike protein's receptor-binding domain may increase infectivity in sperm whales (*Physeter catodon*) [26]. Our work indicating that belugas and dolphins seem to be susceptible to Delta and Omicron variants of SARS-CoV-2 further challenges a scenario whereby SARS-CoV-2 is evolving to specialize solely on humans as hosts.

Myriad factors other than cell entry could govern SARS-CoV-2 infection success in an organismal host, and we cannot make claims about disease course, transmissibility, etc. Nevertheless, the evidence that belugas and dolphins may be permissive hosts to SARS-CoV-2 should warrant increased surveillance efforts in these animals. Beyond the perceived threat to marine mammals themselves, a scenario in which these animals are infected also poses risks to humans owing to potential spillover-spillback events. For example, novel SARS-CoV-2 variants arose within white-tailed deer (*Odocoileus virginianus*) populations and were transmitted back into the human population [41]. The potential of a similar event to happen between humans and marine mammals should not be ignored.

## Data Availability

The data and code are provided in the electronic supplementary material [42].
